# Beneficial Effects of Prebiotic *Saccharomyces cerevisiae* Mannan on Allergic Asthma Mouse Models

**DOI:** 10.1155/2017/3432701

**Published:** 2017-08-01

**Authors:** D. Betty Lew, Christie F. Michael, Tracie Overbeck, W. Scout Robinson, Erin L. Rohman, Jeffrey M. Lehman, Jennifer K. Patel, Brandi Eiseman, Kim S. LeMessurier, Amali E. Samarasinghe, M. Waleed Gaber

**Affiliations:** ^1^Department of Pediatrics, University of Tennessee Health Science Center (UTHSC) and Children's Foundation Research Institute, Le Bonheur Children's Hospital, Memphis, TN, USA; ^2^Department of Pediatrics and Hematology-Oncology, Baylor College of Medicine and Texas Children's Cancer Center, Houston, TX, USA

## Abstract

One of the unmet needs for asthma management is a new therapeutic agent with both anti-inflammatory and anti-smooth muscle (ASM) remodeling effects. The mannose receptor (MR) family plays an important role in allergen uptake and processing of major allergens Der p 1 and Fel d 1. We have previously reported that ASM cells express a mannose receptor (ASM-MR) and that mannan derived from *Saccharomyces cerevisiae* (SC-MN) inhibits mannosyl-rich lysosomal hydrolase-induced bovine ASM cell proliferation. Using a humanized transgenic mouse strain (huASM-MRC2) expressing the human MRC2 receptor in a SM tissue-specific manner, we have demonstrated that ASM hyperplasia/hypertrophy can occur as early as 15 days after allergen challenge in this mouse model and this phenomenon is preventable with SC-MN treatment. This proof-of-concept study would facilitate future development of a potential asthma therapeutic agent with dual function of anti-inflammatory and anti-smooth muscle remodeling effects.

## 1. Introduction

The currently available asthma therapeutics are effective in controlling inflammation but ineffective in controlling pathological changes of airway smooth muscle (ASM) remodeling that can occur at a young age [1, 2]. Therefore, an anti-inflammatory agent that can successfully inhibit ASM remodeling is extremely desirable to reduce asthma-related morbidity and mortality.

There has been a growing interest in the therapeutic use of living microorganisms (probiotics) for many human diseases, including asthma [3–5]. Similarly, prebiotics that are nonliving and indigestible polysaccharides can have beneficial effects on the host. Multiple mannose-rich oligosaccharides are capable of blocking antigen-driven T cell proliferation and antigen uptake and presentation [6, 7]. Acemannan from the Aloe plant induces maturation of dendritic cells [8] and inhibits the proliferative responses of tumor cells by affecting the expression of T lymphocytes [9]. Since the discovery of beer by a Mesopotamian farmer, *Saccharomyces cerevisiae* (Brewer's or Baker's yeast) is quantitatively and economically one of the most important groups of microorganisms exploited by man for food and alcoholic beverages. Mannan derived from *S. cerevisiae* (SC-MN), a prebiotic polymer of mannose (Man9 and a Gln residue, connected by *α* linkages), composes 45% of the cell wall of *S. cerevisiae* [10].

The mannose receptor (MR) family is an intricate part of innate immunity and the homeostatic clearance system [11] and plays an important role in uptake and processing of major allergens Der p 1 and Fel d 1, although the binding sites of these antigens differ [12, 13]. We have previously reported that airway smooth muscle cells express a mannose receptor (ASM-MR) [14] and SC-MN can inhibit bovine ASM cell proliferation induced by endogenous mannosyl-rich MR ligands such as lysosomal hydrolases (*β*-hexosaminidases Hex A and Hex B and *β*-glucuronidase) [15].

In a murine allergic asthma model, mycobacterial acyl chains and mannose groups of lipoglycans have been shown to suppress allergic disease and increase IL-10 secretion from cervical lymph nodes and splenocytes [16]. We have chosen to investigate the effects of SC-MN because mannan from pathogenic microorganisms is capable of eliciting IL-17 production [17]. However, this is not true of SC-MN [17], making it an appealing therapeutic agent as its beneficial effects could be achieved without IL-17-driven inflammation. We have confirmed this finding in our study as well as documented its lack of ability to induce IL-13 production in the lungs of mice despite a potential concern of its ability to regulate dendritic cell function in favor of T_H_2 polarization [18]. Furthermore, SC-MN had no effect on IL-33 that can stimulate group 2 innate lymphoid cells important in fungal allergy [19].

Here, we present evidence that by cloning of the huASM-MRC2 and development of a humanized transgenic mouse model, overexpression of huASM-MRC2 results in accelerated ASM remodeling and SC-MN can offer dual anti-inflammatory and anti-smooth muscle remodeling benefits.

## 2. Materials and Methods

### 2.1. Animals, Cloning huASM-MRC2, and Transgene Construct

All protocols were approved by the University of Tennessee Health Science Center (UTHSC) Institutional Biosafety Committee and Institutional Animal Care and Usage Committee. Wild-type (WT) FVB/NJ and BALB/c were purchased from the Charles River Laboratories or the Jackson Laboratory and housed in a pathogen-free vivarium at the UTHSC. The human ASM-MRC2 was cloned by RT-PCR using primers designed from cDNA of MRC2 and mRNA isolated from human bronchial ASM cells. The human ASM-MR cDNA (5658 bp) is 99% identical to the EST clone KIAA0709 (5641 bp; adult human brain), and the coding region is identical to that of MRC2 cDNA (Endo180, 4639 bp). Full-length cDNA of the huASM-MRC2 coding region was reconstructed, and a transgenic mouse model overexpressing huASM-MRC2 from the smooth muscle-specific SM22*α* gene promotor was developed for the purpose of testing their susceptibility to atopic asthma phenotype. Sense and antisense oligonucleotide primers were designed based on the deposited sequence of SM22*α* gene (5′ region and exon 1; 3892 bp; accession U36589). Hind III sites were added to the 5′ end of both primers for cloning purposes. The primers were used to generate a 502 bp fragment containing the 445 bp SM22 alpha promoter including exon 1 [20] by PCR on genomic mouse DNA. The promoter fragment was ligated to the 5′ end of the full-length coding region of ASM-MR cDNA (4437 bp) in the plasmid pCR 3.1-Uni, and a fragment containing the *β*-globin intron and polyadenylation signal (1.2 kb) was ligated to the 3′ end of ASM-MR cDNA. The 6.2 kb transgene fragment was excised with the restriction endonuclease Pme I and used to generate transgenic mice in the FVB/NJ strain.

The transgenic mice (FVB/NJ) were then backcrossed onto an allergy-prone BALB/c strain, and N10 generation mice of ages 6–8 wk were used for the study. Transgene expression was confirmed using qPCR by Transnetyx Inc. (Memphis, TN, USA): target sequence 3′-cagcgaggacctatgtgctctgccctacacgaggtctacaccatccagggaaactcccacggaaagccg-5′.

For smooth muscle remodeling studies, mice with qPCR signal values between 7.5 and 13.0 were used.

### 2.2. Allergen Sensitization and Challenge

WT BALB/c or humanized transgenic mice were OVA-immunized on days 0 and 14 with 20 *μ*g OVA (Sigma, St. Louis, MO, USA) in Imject® Alum (Pierce, Rockford, IL, USA) [21], i.p.; nonimmunized (sensitized) mice received alum only. Increased serum OVA-specific IgE levels on day 1 versus day 21 were confirmed using ELISA (AbD Serotec, Oxford, UK). Responsive mice (sIgE ≥ 500 ng/ml) were pretreated with control saline or SC-MN (Sigma, prepared for patented use for asthma therapeutic, endotoxin level < 2 EU/ml) 30 min before OVA challenge on days 28, 29, and 30 (100 *μ*g OVA i.n. in 25 *μ*l phosphate-buffered saline (PBS).

### 2.3. Airway Physiology Measurements

To assess isolated thoracic flow, compartmentalized double chambers (Buxco) were used to exclude nasal airway resistance. Conscious spontaneously breathing animals were retrained in the chambers to analyze thoracic-specific airway resistance (sRaw) in response to aerosolized saline or escalating doses of Mch (2.5–25 mg/ml, 1 ml) for 3 min [22]. Readings were taken averaged for 5 min following each nebulization.

### 2.4. Liposome Construction

Liposomes were prepared by the evaporation method using different molar ratios of HSPC : Chol : DSPE-PEG2000 (e.g., 50 : 45 : 5) (Dr. George C. Wood, Department of Pharmaceutical Sciences, UTHSC). Appropriate quantities of lipids and near-infrared fluorescent dye (DiIC18 = DiR) were dissolved in 1 : 9 solvent mixtures of chloroform and methanol. The mixture was evaporated in a Rota-evaporator at 40°C under vacuum at 100 rpm overnight. The lipid film was hydrated at 65°C with HEPES buffer containing 0.15 M NaCl and 10 mM EDTA on the Rota-evaporator at 100 rpm for 2 hr. The resulting fluorescent liposomes were large multilamellar vesicles of approximately 0.8–1.0 *μ*m. Depending on the vesicle size required, liposomes were extruded through stacked nucleopore filters for a total of 6–10 passes, producing a narrow particle size distribution of unilamellar liposomes. Targeting of PEG-maleimide liposomes with the cRGD peptide was performed via covalent coupling through thioester bond formation between the maleimide group of the liposomes and cyclic RGD [23].

### 2.5. Fluorescent Reflectance Imaging (FRI)

On day 32, 48 hr after OVA challenge, mice were anesthetized using isofluorane. Fluorescent liposome (-DiR) (800 nm, 90 *μ*g/ml, 25 *μ*l, each nostril, i.n.) or fluorescent cRGD-liposome (-DiR) (80 nm, 20 *μ*g/ml, 200 *μ*l, i.v.) was administered to the mice. The imaging procedures were performed at 2, 6, and 24 hr postadministration of fluorescent liposome with fixed fiducial markers for optical imaging with different dye concentrations as a standard. The FRI system includes a liquid nitrogen CCD camera (Photometric Chemipro, Roper Scientific, Trenton, NJ) mounted onto a darkbox and a shutter mounted with a filter wheel (Sutter Instruments Co., Lambda 10-2 Optical Filter Changer, Novato, CA) that allows for multiple excitation filters to be controlled through software (Metamorph, Universal Imaging, Downingtown, PA). An excitation filter centered at 710 nm (Chroma Technology Corp., Rockingham, VT) and a 3-cavity narrow bandpass filter (10 nm spectral width) (780 nm at peak wave) (Andover, Salem, NH) were used to visualize the near-infrared light. The biologic autofluorescence region is approximately 400–600 nm wavelength, and therefore, DiR signals were able to be detected without interference by autofluorescence.

### 2.6. Bronchoalveolar Lavage (BAL) and Lung Tissue Collection

Bronchoalveolar lavage was performed with 0.5 ml of warm PBS (37°C) under ketamine/xylazine sedation (100 mg/kg each). Cell count was performed by cytospin, and cell-free BAL fluid (BALF) was stored at −80°C. Total lung tissue was homogenized in 1 ml of PBS containing protease inhibitor cocktail (Sigma) on ice. Protein was quantified by using a Bradford Protein Assay kit (Thermo Fisher). Both BAL fluids and lung homogenates were analyzed for cytokines by a Quantikine ELISA kit from R&D systems according to the manufacturer's instructions. Muc5ac in BAL fluid was analyzed by an ELISA kit (USCN Life Sci. Inc., Houston, TX) according to the manufacturer's instructions.

### 2.7. IL-10 ELISPOT Analysis

Naïve mice were fed once with SC-MN (80 mg/kg) or saline, via gavage. Eighteen hours later, BAL cells and unselected splenocytes (1 × 10^5^ cells/well of each cell type) were plated onto a Millipore microtiter well plate for IL-10 ELISPOT analysis. Cells were stimulated with or without SC-MN (1 mg/ml) and cultured for 72 hrs at 37°C in CO_2_ incubator (5% CO_2_/95% air). The IL-10-producing cells were identified following the manufacturer's instructions (BD Pharmingen) and sent out to BD Biosciences analysis service.

### 2.8. Histology, Immunohistochemistry, Histochemistry, and Analysis

Mice were sacrificed (cervical dislocation under anesthesia) and perfused with 4% paraformaldehyde. Paraffin-embedded trachea and lung tissue sections were cut at 10 *μ*m for H&E staining, immunohistochemistry was performed for smooth muscle isoactin, and Alcian blue-periodic acid Schiff (PAS) stains were used for mucin-containing goblet cells. Smooth muscle *α*-isoactin was probed with the *α*-smooth muscle actin antibody clone 1A4 according to the manufacturer's instruction using a kit (Sigma). All slides were scanned using the ScanScope® XT at 0.25 *μ*m/pixel resolution (~400x magnification). Trachea cross sections were analyzed for the trachealis muscle area at day 38, 45, or 52. The small airway smooth muscle area was analyzed by selecting 3–5 small airway branches with cross-sectional cut (120–300 *μ*m calibers) at day 45. Data collection and analyses were performed blinded (*n* = 5-6 mice) using the fiduciary marker 200 *μ*m average (i.d.) airway caliber and 31,416 *μ*m^2^ luminal area. Each data point represents an averaged value from one mouse.

### 2.9. Statistical Analysis

All animal experiments were repeated three times. Data are expressed as mean or median ± 95% CI (for PC200R, provocative methylcholine concentration effecting a 200% increase in airway resistance) or mean ± SD (or SEM) and analyzed by the Kruskal-Wallis test followed by post hoc tests for unpaired data or one-way analysis of variance followed by Dunnett's post hoc test using Prism 6 software (GraphPad, San Diego, CA). For FRI data, software (Metamorph, Universal Imaging, Downingtown, PA) was used to quantify the intensity of fluorescence. *P* values less than 0.05 were considered statistically significant.

## 3. Results

### 3.1. Anti-Inflammatory Effects of SC-MN on OVA-Allergic WT BALB/c Mice

**Figure 1:**
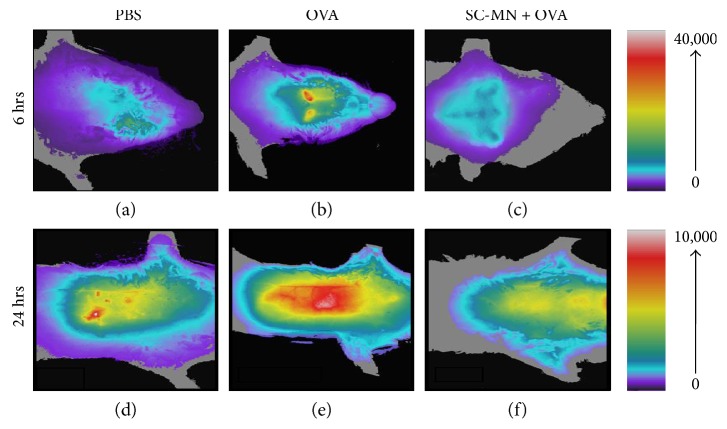
Homing of cyclic RGD-liposomes to cervical lymph nodes and lungs in allergic asthmatic mice. Cyclic RGD- and DiR-labeled liposomes were injected intravenously into OVA-allergic BALB/c mice. (a, d) PBS-challenged; (b, e) OVA-challenged; (c, f) mannan pretreatment (1 mg, i.n., 30 min before each of the three OVA challenges). FRI images were acquired 2, 6, and 24 hr postinjection. The intensity of the signals in the cervical lymph nodes (c) and lungs (f) in SC-MN-pretreated mouse was markedly diminished compared to that in the OVA-challenged mouse (b, e). Signals in the lungs were detected at the 24 hr time point (d–f). The results are representative of 2 separate experiments with similar results.

**Figure 2:**
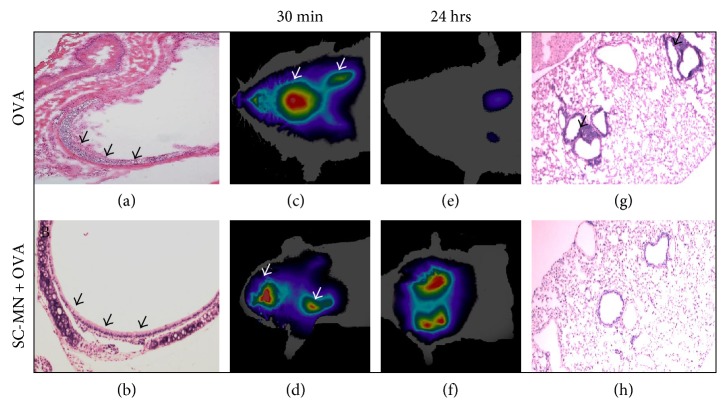
Effect of SC-MN on OVA-induced airway pathology in BALB/c mice. Upper panels: OVA-allergic BALB/c mouse. Lower panels: SC-MN-treated allergic BALB/c mouse (1 mg, i.n., 30 min prior to each OVA challenge). (a) H&E staining of the trachea shows epithelial denudation (arrow) compared to that of the intact epithelium in SC-MN-treated mouse (b). (c–f) FRI of mice 30 min (c, d) and 24 hrs (e, f) post i.n. administration of liposome. Notice the increase in central airway signals (arrow head) with less lung deposition (c) and faster clearance (arrow) (e) in untreated allergic mouse compared to the SC-MN-treated mouse (d, f). Images of the abdomen showed no signals in the stomach. Color intensity: red > yellow > green > blue. The FRI images are representative of 2 separate experiments with similar results. (g) Peribronchial cellular infiltration in H&E lung sections compared to the paucity of peribronchial cellular infiltration in SC-MN-treated mouse (h), representative of 3 separate experiments. Final magnification: 100x.

For a global view of anti-inflammatory effects of SC-MN, we employed a whole-body imaging system to identify sites of inflammation; fluorescent cyclic arginine-glycine-aspartic acid- (cRGD-) labeled liposomes were used to detect integrin upregulation as a marker of inflammation. Intravenously (i.v.) administered cRGD first homed to the cervical lymph nodes and then to the lungs in the OVA-allergic mice, which was partially mitigated by intranasally (i.n.) administered SC-MN treatment. The upper limit of signals from lymph nodes was 4 times higher than that of the signals in the lungs. Cyclic RGD homing to the cervical lymph nodes and lungs was prominent compared to the control or SC-MN treatment in mice (Figures 1(a), 1(b), 1(c), 1(d), 1(e), and 1(f)), suggesting that SC-MN treatment suppressed inflammation in the draining lymph nodes and lungs. Intranasal pretreatment with SC-MN (1 mg, 30 min before each of three OVA challenges) inhibited allergic airway inflammation allowing i.n. administered liposome to reach the lungs of SC-MN-pretreated allergic mice, compared to the little deposition of liposome in the lungs of control allergic mice (Figures 2(a), 2(b), 2(c), 2(d), 2(e), 2(f), 2(g), and 2(h)). This finding is presumably due to mucociliary clearance of the liposome particles in the inflamed airways of untreated allergic mice. Allergic mice pretreated with SC-MN showed marked inhibition of cellular infiltration (Table 1).

Similar to the report on lipoglycan [16], SC-MN can stimulate IL-10 regulatory cytokine as shown in Table 2.

To confirm that SC-MN does not cause potentially harmful effects of IL-17 production [17], T_H_2 polarization [18], or IL-33 production that can stimulate ILC2 cells [19], we have measured IL-13, IL-17, IL-22, and IL-33 in BALF and supernatants of lung homogenates in naïve BALB/c mice after daily SC-MN (or saline) for 3 consecutive days. There was no increase in any of the abovementioned cytokines in SC-MN- treated naïve BALB/c mouse lung homogenate supernatants: IL-13, 115 ± 82 versus 51 ± 11; IL-17, 125 ± 31 versus 123 ± 28; IL-22, 8 ± 7 versus 8 ± 9; and IL-33, 5792 ± 2012 versus 5553 ± 1257 pg/mg protein (saline versus SC-MN group, resp., *n* = 7 mice). Similarly, there was no discernable difference in any of these cytokines measured in the BALF of these two groups.

### 3.2. Effect of SC-MN on Airway Mucin

**Figure 3:**
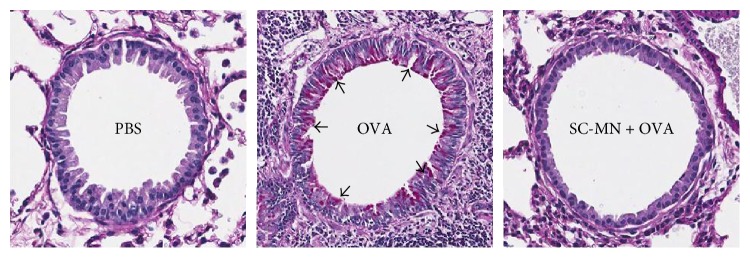
Effect of SC-MN on airway mucin in allergic mice. PAS staining visualizes neutral mucins (magenta material indicated by arrows) from tissue morphology at day 31. Intranasally administered SC-MN effectively blocked the ovalbumin- (OVA-) induced increase in mucin in small airway tissue sections. Similar results were obtained at day 45.

**Figure 4:**
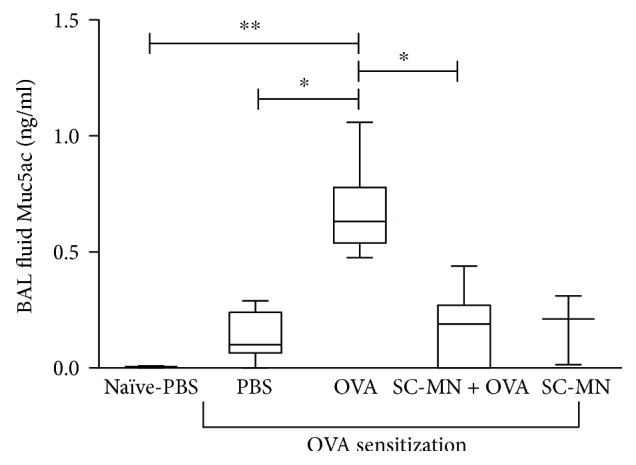
SC-MN effectively inhibits Muc5ac protein levels in BAL fluid of allergic mice. BAL fluid was obtained at day 45. Results are mean ± SEM (*n* = 3 for naïve control with PBS challenge); *n* = 7 for OVA-sensitized groups). ^∗∗^*P* < 0.01 versus PBS-challenged naïve mice; ^∗^*P* < 0.05 versus PBS-challenged allergic mice or SC-MN-treated allergic mice.

Mucous hypersecretion from goblet cells is a hallmark of asthma, which can lead to airway obstruction and increased morbidity [24]. The effect of SC-MN on airway neutral mucin was examined by PAS staining in the midlung sections (Figure 3); stored mucin within the epithelium was inhibited by SC-MN in allergic mice (59–81% inhibition, *n* = 6). Muc5ac protein levels in BAL fluid were significantly elevated in allergic mice that were effectively inhibited by SC-MN treatment (Figure 4).

### 3.3. Inhibition of AHR by SC-MN (Intranasal) in WT Allergic BALB/c Mice

**Figure 5:**
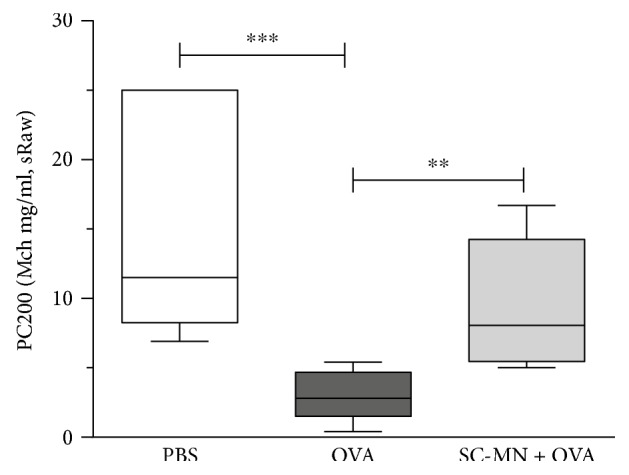
Inhibition of AHR by intranasal SC-MN in WT BALB/c OVA-allergic mice. All mice were OVA-sensitized and OVA-challenged, and some mice were pretreated with SC-MN 30 min before each challenge with OVA. Thoracic PC200R (sRaw), median ± 95% CI. ^∗∗^*P* < 0.05 versus OVA challenge with saline pretreatment. ^∗∗∗^*P* < 0.001 versus PBS challenge with saline pretreatment.

Based on preliminary dose response studies (6–60 mg/kg), we have selected 45 mg/kg as an optimum efficacious SC-MN dose targeting ASM cells and thoracic sRaw parameter. Mice pretreated with SC-MN (45 mg/kg, i.n., 30 min before OVA challenges) showed significantly blunted AHR, measured by PC200R (provocative Mch concentration effecting a 200% increase in airway resistance), compared to untreated allergic mice (OVA group) (Figure 5).

### 3.4. Accelerated ASM Remodeling in a huASM-MRC2 Mouse Model

**Figure 6:**
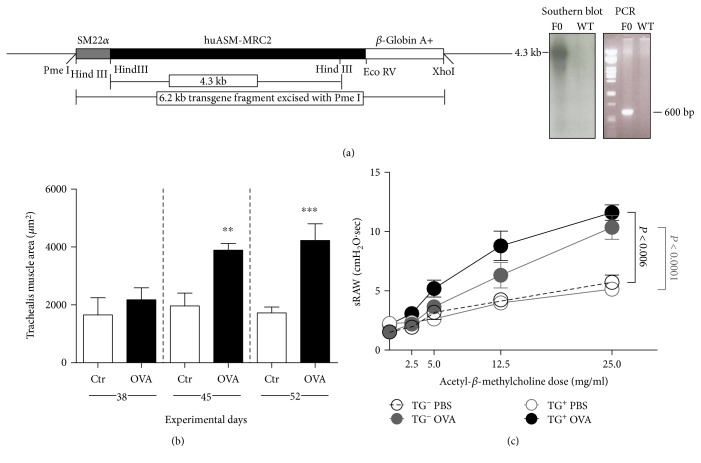
Accelerated large airway smooth muscle remodeling in a huASM-MRC2 transgenic allergic asthma mouse model. (a) Schematic of the construct used to generate huASM-MRC2 transgenic (Tg+) mice. The resulting transgenic founders were confirmed by Southern blot, probing Hind III-digested DNA with a 1 kb fragment of the huASM-MRC2 gene (hybridizing to a ~4.3 kb DNA fragment), and PCR analysis using huASM-MRC2 oligonucleotide primers (600 bp product). (b) Mice were OVA-sensitized and OVA-challenged. Control mice received OVA sensitization and PBS challenge. Histology was performed on day 38, 45, or 52. Results are mean ± SD (*n* = 6–11). ^∗∗^*P* < 0.01. ^∗∗∗^*P* < 0.001. (c) Thoracic sRaw in allergic huASM-MRC2 transgenic mice (N10, backcrossed onto BALB/c). A shift of the curve to the left indicates Mch sensitivity (AHR). Numerous breaths were rejected from the automated data acquisition due to shallow labored breathing at Mch 25 mg/ml. Data are mean ± SD (*n* = 6).

Groneberg et al. have demonstrated airway smooth muscle remodeling in OVA-allergic BALB/c mice using a long-term, labor-intense protocol [24]. We have developed a mouse model to substantially shorten the process from a total of 163 days to 45 days (Figures 6(a) and 6(b)). Trachealis muscle mass in OVA-sensitized and OVA-challenged mice remained unchanged in WT (data not shown) and Tg-negative littermate control groups at day 52 (Figure 6(b)). The transgenic allergic mice showed AHR by a shift to the left of the thoracic airway resistance curve (Mch sensitivity) compared to Tg-negative littermate control allergic mice (Figure 6(c)). At the maximum dose of Mch (25 mg/ml), numerous breaths of Tg+ allergic mice were rejected from data acquisition due to shallow labored breathing.

### 3.5. Inhibition of Large and Small Airway Smooth Muscle Remodeling and AHR by SC-MN in Smooth Muscle-Specific huASM-MRC2 Transgenic Allergic Mice

**Figure 7:**
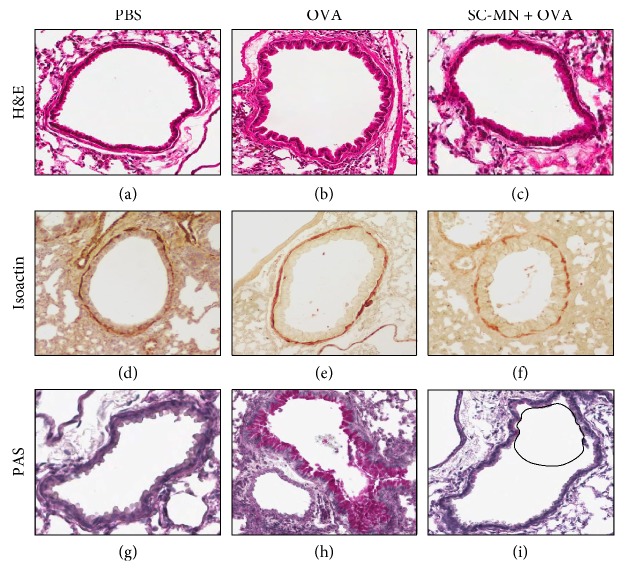
Intranasal mannan blocked the OVA-induced increase in small airway smooth muscle as well as in cellular infiltration, in allergic transgenic mice. OVA-sensitized mice treated with SC-MN prior to OVA or PBS challenge. Representative sections show tissue morphology at day 45, visualized by H&E staining (midlevel left lung). (a) Saline + PBS (control); (b) saline + OVA; (c) SC-MN 45 mg/kg + OVA. Isoactin staining shows organized and thickened ASM in the OVA group (e) compared to the control (d) and SC-MN-treated mice (f). PAS staining shows magenta neutral mucin staining in OVA-sensitized and OVA-challenged mice (h).

**Figure 8:**
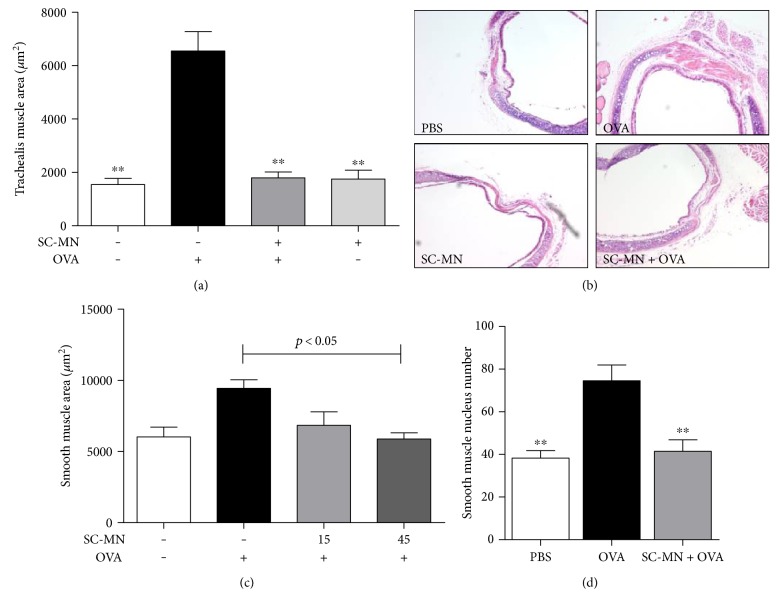
Intranasal SC-MN pretreatment inhibited the OVA-induced increase in small airways ASM, trachealis muscle mass, and number of ASM nuclei of small airways in huASM-MRC2 mice. (a) SC-MN inhibited the trachealis muscle remodeling induced by OVA (day 52) (*n* = 6–11); (b) H&E staining of trachea cross section; (c) small airway ASM area (day 45) (*n* = 5-6); (d) SC-MN inhibition of the increased small airway ASM nuclei in allergic transgenic mice (*n* = 4–7). ^∗∗^*P* < 0.01 versus OVA-challenged group with saline pretreatment.

**Figure 9:**
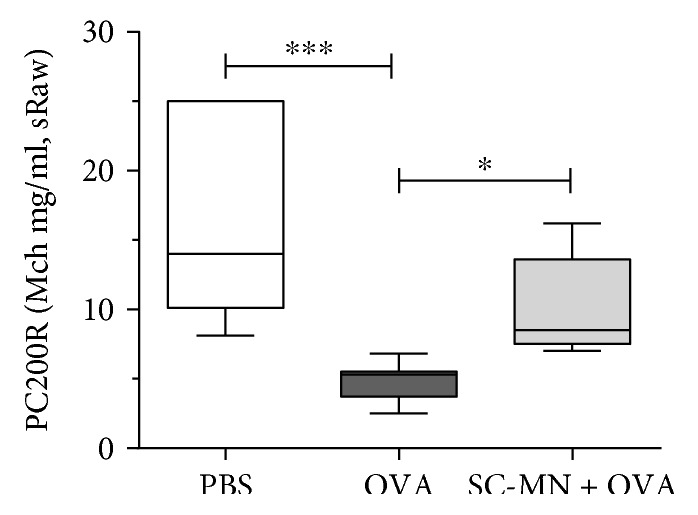
Inhibition of AHR by intranasal SC-MN in huASM-MRC2 transgenic OVA-allergic mice. All mice were OVA-sensitized and OVA-challenged, and some mice were pretreated with SC-MN 30 min before each challenge with OVA. Thoracic PC200R (sRaw), median ± 95% CI. ^∗^*P* < 0.05 versus OVA challenge with saline pretreatment. ^∗∗∗^*P* < 0.001 versus PBS challenge with saline pretreatment (*n* = 7 in each group).

The level of *β*-Hex in the BAL fluid of allergic mice is 3 times higher than that of naïve counterparts (data not shown). To examine the impact of blocking ASM-MR/*β*-Hex-mediated remodeling using SC-MN, the universal allergen OVA was used to induce general allergic inflammatory states in transgenic mice. SC-MN not only significantly inhibited large and small airway smooth muscle mass (Figures 7 and 8) but also inhibited the increase in the number of small airway smooth muscle nuclei (Figure 8(d)). SC-MN treatment (45 mg/kg) significantly restored the decrease in PC200R seen in transgenic allergic mice (Figure 9).

## 4. Discussion

In this study, we have demonstrated the efficacy of SC-MN in suppressing inflammation including stored and secreted mucin and airway hyperreactivity in WT BALB/c allergic mice. In addition, our newly developed transgenic huASM-MRC2 mouse model enabled us to demonstrate ASM remodeling in a reasonable experimental time period, compared to other models [25], that was successfully modified by SC-MN. Airway hyperreactivity was inhibited in this model as well. Airway remodeling in this model showed two elements: (1) an accelerated increase in ASM mass and (2) proliferation of ASM, indicated by ASM nucleus count. Both of these elements were effectively inhibited by SC-MN, consistent with antimitogenic/antiproliferative benefits seen in bovine ASM cells [15].

The mannose receptor blocker, mannan, elicits structure- and source-dependent differential responses in dendritic cells [26]. For instance, the response to monomeric engagement of mannan-MR stimulates IFN-*γ* and IL-12, aiding host immunity against pathogens and regulating T_H_2 microenvironment. On the other hand, mycobacterial lipoglycans have lipid appendages tending to aggregate MRs and suppress IFN-*γ* and IL-12 production, causing immunosuppression in host. The SC-MN preparation used in this study lacks acyl moieties (confirmed by Avanti Polar Lipids Inc. analytical service). Therefore, a higher dose of SC-MN compared to that of lipoglycans [16] was required to exert an anti-inflammatory effect. Regarding the mechanism of anti-inflammation, a single dose of oral SC-MN to naïve FVB/NJ mice, followed by *in vitro* stimulation with SC-MN for 72 hr, resulted in IL-10 production by BAL cells and splenocytes. Sayers et al. also reported a robust induction of IL-10 by lipoglycan from splenocytes and mediastinal lymph nodes from allergic mice using anti-CD3/CD28 stimulation *in vitro* [16]. However, transgenic overexpression of IL-10 can cause lung fibrosis, suggesting potential harmful effects of excess IL-10 beyond its beneficial immunoregulatory role [27]. There are multiple advantages of SC-MN over mannan from other sources: SC-MN is water-soluble and the molecular mass is relatively small (36 kD) compared to that of MN from *Aloe barbadensis* (1000 kD) [8]. Additionally, acemannan and MN from many other sources have beta linkages that are likely to be antigenic [28].

Our findings highlight an important role of huASM-MRC2 (a phylogenetically conserved pattern recognition receptor) that is encoded within chromosome17q23.2 near ORMDL3 17q12-21 of which pathogenic variants increase the risk of asthma [29]. Therefore, members of the MR family, endogenous ligands, and receptor blockers can be exploited for the treatment of human asthma and other diseases. Glycoprotein ligands for the MR family members may be distinct with predilection to bind to different domains [30], and specific function of each member may be diverse depending on the type of ligand, cell, and tissue. For instance, complex protease-resistant endogenous glycoproteins bind to MR at the high-affinity binding domains (CTLD4-5) while SC-MN binds through CTLD4–8 [14]. Single-nucleotide polymorphisms (SNPs) of MRC1 have been associated with asthma in Japanese and African-American populations [31]. Investigation to determine possible association between MRC2 SNPs and asthma is underway. It is reasonable to assume that a family of receptors with such an intricate role in innate and adoptive immunity as well as homeostatic clearance could play an important role in immunomodulation. Recently, gene variants of glycoproteins, affecting serum levels of respective glycoproteins, have been reported as important factors in asthma [32]. Regarding glycoprotein endogenous ligands for MR, *β*-hexosaminidase levels are significantly elevated in serum of severe asthmatic patients [33]. There is also a growing interest in immunoregulatory capacity of glycans and glycan-binding proteins such as galactins, selectins (C-type lectins, MR is a ligand for L-selectin) [34], and siglecs [35].

In conclusion, the prebiotic mannose receptor blocker SC-MN is a promising agent that can render dual benefits in asthma: anti-inflammatory and anti-smooth muscle remodeling at the level of both large and small airways.

## Figures and Tables

**Table 1:** Effect of intranasally administered SC-MN on airway inflammation in WT BALB/c allergic mice.

Aerosol challenge	Total cell number/ml	Macrophages	Lymphocytes	Eosinophils	Neutrophils
PBS	326,000 ± 6000	245,000	55,000	9780	6520
OVA	530,000 ± 30,000^∗^	260,000	196,000	27,000	11,000
SC-MN + OVA	242,000 ± 18,000	138,980	96,960	4040	2020

Mice pretreated with SC-MN (i.n.) showed marked reduction in total BAL cell numbers compared to the OVA group. The decrease is also reflected in all four cell types analyzed. Results are mean ± SEM for total cell numbers in BALF and mean differential cell counts (*n* = 3). ^∗^*P* < 0.05, OVA group versus PBS or SC-MN + OVA group.

**Table 2:** A single oral administration of SC-MN increases IL-10 producing cell numbers in naïve mice.

Specimen	Gavage feeding	Immunospot	Mean spot sizes
BAL	Saline	163 ± 161	0.0052 ± 0.0022
BAL	SC-MN	586 ± 211^∗^	0.0060 ± 0.006
Splenocytes	Saline	35 ± 13	0.0133 ± 0.0064
Splenocytes	SC-MN	427 ± 221	0.0077 ± 0.0009

Results are mean ± SEM (*n* = 3). We observed that administering SC-MN to mice increased the number of IL-10-producing cells in the BAL (^∗^*P* < 0.05). A similar trend was seen for splenocytes from SC-MN-treated mice.

## Abbreviations

AHR: Airway hyperreactivity
ASM: Airway smooth muscle
BAL: Bronchoalveolar lavage
*β*-Hex A and B: *β*-Hexosaminidase A and B (aka NAGA (N-acetyl-*β*,D-glucosaminidase), NAG, and NAGase)
CCD: Charge-coupled device
CTLD: C-type (calcium-dependent binding) lectin-like domain
cRGD: Cyclic arginine-glycine-aspartic acid (D)
DiR: DiIC18-indotricarbocyanine iodide-phycoerythrin
DSPE-PEG: 1,2-Distearoyl-sn-glycero-3-phosphoethanolamine-polyethylene glycol
FRI: Fluorescent reflectance imaging
huASM-MRC2: Mannose receptor C-type 2 cloned from human airway smooth muscle cells
MRC1: C-type mannose receptor 1 (CD206, macrophage mannose receptor)
MRC2: C-type mannose receptor 2 (CD280, Endo180, urokinase plasminogen activator receptor-associated protein)
OVA: Ovalbumin
PC200R: Provocative methacholine (methylcholine) concentration effecting a 200% increase in airway resistance
PC: Phosphatidylcholine
SC-MN: Mannan derived from *Saccharomyces cerevisiae.*
